# Active learning and sector-specific simulations mitigate the first-month injury risk in young workers

**DOI:** 10.3389/fpubh.2026.1767451

**Published:** 2026-02-09

**Authors:** Vedat Caner

**Affiliations:** Department of Occupational Health and Safety, Vocational School, Istanbul Beykent University, Istanbul, Türkiye

**Keywords:** accident analysis, active learning, occupational health and safety, safety training, vocational education, young workers

## Abstract

**Introduction:**

Occupational health and safety education is a critical preventive strategy for mitigating workplace accidents, particularly among young workers who face disproportionate risks during their transition from school to work. This study aims to evaluate the effectiveness of current educational interventions and analyze sectoral accident patterns to inform curriculum development.

**Methods:**

A systematic review was conducted synthesizing data from 32 international studies published between 2004 and 2025. The study employed a mixed-methods design, integrating quantitative accident statistics with qualitative assessments of pedagogical models to correlate training methods with safety outcomes.

**Results:**

The analysis reveals that active learning methods significantly improve safety motivation and internal locus of control compared to traditional passive instruction. Crucially, accident data identifies a “first-month vulnerability,” with up to 67% of injuries in vocational settings occurring within the initial weeks of employment. Furthermore, sectoral comparisons demonstrate that generic safety curricula fail to address specific lethal risks, such as falls in construction or transport-related injuries in healthcare.

**Discussion:**

Current vocational training models are insufficient for ensuring early-career safety. Sustainable injury prevention requires a paradigm shift in curricula from generic compliance rules to sector-specific simulations and mandatory transition phases that mimic real-world workplace pressures.

## 1 Introduction

Occupational Health and Safety (OHS) is a fundamental human right and a critical component of sustainable economic development. However, despite rigorous regulations and technological advancements, occupational accidents remain a global plague, disproportionately affecting young and inexperienced workers (1, 2). The transition from the protected environment of vocational schools to the dynamic and often hazardous reality of industrial workplaces represents a period of significant vulnerability. Statistics indicate that young workers are injured at higher rates than their older counterparts, largely due to a lack of experience and inadequate safety training during their formative years (2, 3).

The role of Vocational Education and Training (VET) institutions is pivotal in mitigating these risks. Ideally, VET should equip students not only with technical skills but also with the “safety competence” required to navigate workplace hazards (4). However, recent studies suggest a disconnect between the theoretical OHS curriculum taught in schools and the practical, situational risks encountered in sectors such as construction, mining, and healthcare (5, 6). For instance, traditional teaching methods often fail to address the complexity of real-world decision-making, leading to a “safety capability gap” among graduates (7).

Furthermore, the “one-size-fits-all” approach to OHS education is increasingly challenged by sectoral data. The risk profile of a construction site, where falls from heights are prevalent (8), differs vastly from the biological hazards faced by healthcare workers (9) or the machinery risks in mining (10). Therefore, educational curricula must be responsive to these specific accident patterns to be effective.

This study aims to synthesize evidence from 32 recent international studies to evaluate the effectiveness of various OHS educational interventions and analyze sectoral accident data to inform curriculum development. Specifically, this review addresses the following research questions:

- *RQ1*: How do different OHS pedagogical methods (e.g., ZeroSicks, active learning) impact students’ safety awareness and injury rates?
- *RQ2*: How should sectoral accident analysis (mining, construction, health) shape the content of OHS curricula?
- *RQ3*: What is the role of school-based OHS education in reducing the “first-month” injury risk for young workers?

Operational Definitions To ensure clarity regarding the scope of this review, the following operational definitions are adopted: “Vocational Education and Training (VET)” refers to educational programs that prepare students for specific trades (e.g., vocational high schools, technical institutes). “OHS Education” encompasses both standalone safety training interventions (e.g., First Aid courses) and broader vocational curricula that integrate safety components. This review considers both types of programs to evaluate the overall “safety competence” of young workers transitioning to the labor market.

## 2 Methods

### 2.1 Research design

This study is designed as a systematic mixed-methods review utilizing a convergent integrated synthesis approach. This design was selected to allow for the integration of quantitative findings (e.g., accident statistics, injury rates) with qualitative insights (e.g., pedagogical models, safety culture assessments). While a quantitative meta-analysis was initially considered, it was determined to be infeasible due to the significant heterogeneity of outcome measures across the included studies (ranging from Injury Rate Ratios to qualitative perception scores). Therefore, a narrative synthesis of the quantitative data was combined with a thematic analysis of the qualitative data.

### 2.2 Data sources and search strategy

The systematic search was conducted in four major scientific databases: Scopus, Web of Science, PubMed, and Science Direct. The search strategy followed the PRISMA guidelines. Boolean operators were used to combine keywords related to vocational education (“OHS education,” “vocational training,” “active learning”) with keywords related to safety outcomes (“young workers,” “accident analysis,” “first month accidents”). The review included peer-reviewed articles published between 2004 and 2025 to capture both historical baselines and recent digital interventions.

### 2.3 Study selection and eligibility criteria

Studies were selected based on the following inclusion criteria:

1. *Relevance:* Studies evaluating specific OHS training programs in vocational or workplace settings; (4, 11–13).
2. *Sectoral focus:* Studies providing distinct accident data for high-risk sectors (construction, mining, health); (5, 8, 10, 14).
3. *Target population:* Research focusing on young workers (15–24 years) or vocational students; and (2, 3, 6).
4. *Methodological rigor:* Peer-reviewed articles utilizing valid statistical methods (e.g., SEM, regression) or rigorous qualitative analysis.

**Figure 1:**
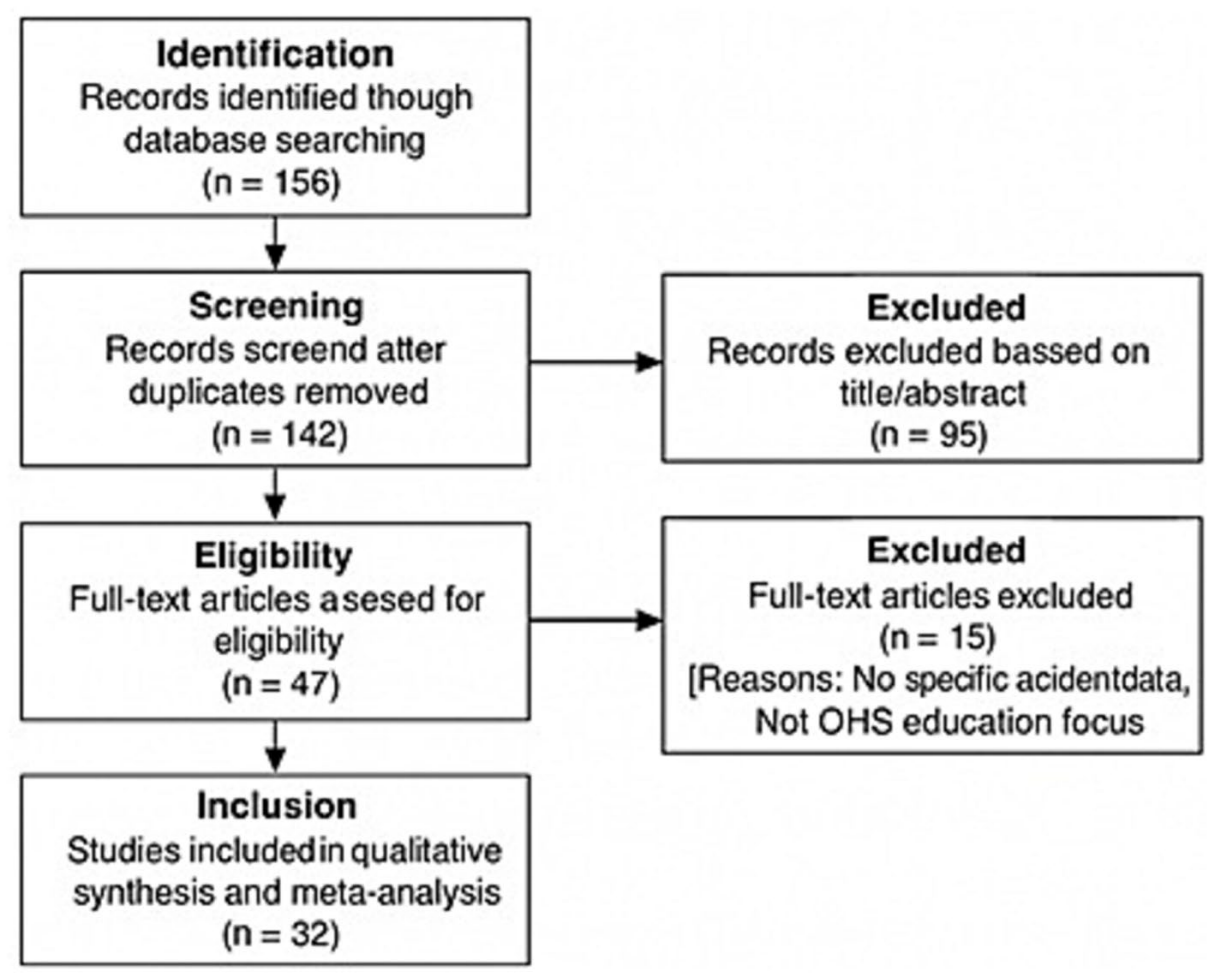
PRISMA flow diagram of the study selection process.

The selection process followed a two-stage screening (title/abstract and full-text) as detailed in the PRISMA flow diagram (Figure 1).

The screening was conducted independently. Any disagreements regarding the inclusion of a study were resolved through discussion and consensus between the researchers. Of the 142 records screened after duplicate removal, 95 were excluded at the title/abstract stage primarily due to irrelevance (e.g., studies not focusing on OHS education or lacking young worker data). Subsequently, 15 full-text articles were excluded because they did not provide specific sectoral accident data or sufficient methodological detail. The review protocol was not registered in PROSPERO prior to the commencement of the study.

### 2.4 Data extraction protocol

Data extraction was performed using a standardized coding form developed in Microsoft Excel to ensure consistency. For each included study, the following variables were extracted:

- *Bibliographic details*: Author(s), year of publication, and country of origin.
- *Methodological characteristics*: Study design (e.g., prospective cohort, cross-sectional, qualitative case study), sample size, and data collection instruments.
- *Sectoral context*: The specific industry (Construction, Mining, Healthcare, etc.) and the nature of the work environment.
- *Intervention details*: Description of the educational approach (e.g., passive lecture *vs.* “ZeroSicks” active model, VR simulation).
- *Outcome measures*: Quantitative metrics (Injury Rate Ratios—IRR, accident frequency, severity indices) and qualitative outcomes (safety motivation scores, locus of control changes). To ensure reliability, the extraction was conducted iteratively, with a random sample of 20% of the papers cross-checked to verify coding accuracy.

### 2.5 Quality assessment

Given the inclusion of quantitative, qualitative, and mixed-method studies, the methodological quality of the selected papers was assessed using the Mixed Methods Appraisal Tool (MMAT), version 2018. This tool allows for the concomitant appraisal of diverse study designs. Studies were evaluated based on criteria such as the appropriateness of the sampling strategy, the validity of measurements (for quantitative studies), and the coherence between data sources and interpretation (for qualitative studies). Studies failing to meet the core quality criteria (e.g., lack of clear research questions or insufficient data to support conclusions) were excluded during the full-text review phase to ensure the reliability of the synthesis.

### 2.6 Data synthesis and analysis

As noted in the research design, statistical pooling (meta-analysis) was not conducted due to the methodological diversity of the included studies (which ranged from quantitative injury rate calculations to qualitative safety climate perceptions). Therefore, a Convergent Integrated Design was employed for data synthesis:

1. *Thematic synthesis*: Qualitative findings regarding pedagogical methods (e.g., the effectiveness of active learning) were coded into themes (e.g., “Internalization of safety,” “Simulation efficacy”).
2. *Narrative synthesis of quantitative data*: Statistical findings regarding accident causes were grouped by sector. Accident frequency patterns, particularly regarding the “first-month effect,” were analyzed to identify temporal trends.
3. *Triangulation*: The qualitative themes on education gaps were mapped against the quantitative data on accident causes to identify disconnects (e.g., matching the high prevalence of “falls” in construction data with the lack of “working at heights” training in curricula).

## 3 Results

A representative summary of the key studies included in this review is presented in Table 1. These studies were selected to illustrate the geographical spread (Europe, Asia, Middle East), sectoral variety (Mining, Construction, Health), and methodological diversity (Quantitative *vs.* Qualitative) of the analyzed literature.

**Table 1:** Summary of key studies included in the systematic review (selected representative sample).

Author(s) (year)	Country	Sector/Context	Methodology	Key findings/Contribution
Boini et al. (3)	France	General (young workers)	Prospective cohort	OHS education during schooling reduces injury risk by 50% (*IRR* = 0.51). First aid training further lowers risk.
Ismara et al. (12)	Indonesia	Vocational education	SEM/ZeroSicks model	Active learning models (“ZeroSicks”) significantly improve safety awareness and behavior (*t*-*value* = 2.431).
Aliabadi et al. (10)	Iran	Mining	Bayesian network	Safety training is the most influential factor (44.76%) on accident severity, surpassing experience.
Zermane et al. (8)	Malaysia	Construction	Statistical analysis	Falls from heights account for 32% of fatalities. General workers are the most vulnerable group (60%).
Khairallah et al. (9)	Lebanon	Healthcare	Retrospective study	Transport accidents (*OR* = 5.92) cause more severe injuries than needle-sticks (*OR* = 0.008) among hospital staff.
Tuganishuri et al. (22)	S. Korea	General industry	Multinomial logistic regression	67% of accidents occur within the first month of employment.
Talib et al. (7)	Malaysia	TVET education	Fuzzy Delphi	Current OHS curricula lack focus on “safety motivation” and “communication.”
Baraza et al. (1)	Spain	Mining	Statistical analysis	Smaller companies have significantly higher fatal accident rates (*FAR* = 51.11%) than larger firms.

### 3.1 Effectiveness of educational interventions (RQ1)

The review reveals that structured and active OHS education significantly reduces accident rates and improves safety behavior.

- *Impact on injury rates:* A prospective cohort study involving 755 young workers in France demonstrated that those who received OHS education during schooling had a “two times lower risk” of workplace injuries compared to those who did not (*IRR* = 0.51) (3). Similarly, specific “first aid at work” training was associated with a further reduction in injury risk (*IRR* = 0.68) (3).
- *Pedagogical methods:* Traditional lecturing is less effective than active learning. In Indonesia, the implementation of the “ZeroSicks” method (Zero Accident and Sickness) in vocational high schools resulted in “excellent” safety awareness scores among students, confirmed by Structural Equation Modeling (SEM) analysis (*t*-*value* = 2.431) (12). In Finland, the “Attitude to Work” intervention, which utilized active learning techniques and safety skills training, showed positive associations with students’ safety motivation (*b* = 0.37) and internal safety locus of control (4). Additionally, training programs specifically designed to optimize competence have been shown to be crucial in the effective implementation of OHS practices (15).
- *Gaps in implementation:* Despite these successes, gaps remain. In Malaysia, a needs analysis using the Fuzzy Delphi method indicated that current curricula are insufficient and require improvements in “safety motivation” and “safety communication” components (7). In Bhutan, while trainees showed high awareness of PPE usage (mean rating 4.09), the lack of management support and monitoring hindered the practical application of these skills (16). In Sudan, a lack of training was identified as a key factor in accidents involving defective equipment in the engineering sector (17).

### 3.2 Sectoral accident analysis and curriculum implications (RQ2)

The analysis of accident data highlights that generic OHS training is insufficient; curricula must address specific sectoral risks.

- *Construction:* Falls from heights and being struck by objects are the dominant causes of fatalities. In Malaysia, falls accounted for 32% of fatal accidents (8). A meta-analysis of the Greek construction industry identified “worker training deficiencies” as a top-level accident factor, specifically linking lack of training to falls and slippage (5). In South Korea, trend analysis revealed that construction accidents often stem from inadequate safety management in smaller projects (18).
- *Mining:* In the mining sector, Bayesian network analysis identified “safety training” as the single most influential factor on accident severity (44.76% influence), surpassing even experience (10). In Spain, mining accidents were found to be 14 times more fatal than the average of all sectors, with machinery handling being a critical risk factor (1).
- *Healthcare:* Unlike heavy industries, healthcare risks are often underestimated. A study in Lebanon found that while needle-stick injuries are frequent (50.25%), they are less severe than transport-related injuries among healthcare workers (*OR* = 5.927) (9). This implies that healthcare OHS curricula should expand beyond biological hazards to include commuting safety and ergonomic risks for nurses (9).
- *Manufacturing and general industry:* In Italy, extreme temperatures were found to increase injury risks, particularly for young workers in the construction and trade sectors (19). In Portugal, machinery accidents remain a significant cause of occupational injuries, highlighting the need for technical safety training (14). Modern approaches, such as optimized deep learning models, are now being used to contextualize injury severity from accident reports, providing deeper insights for prevention (20). In Sudan, “caught in or between objects” was the most frequent accident type (17). Similarly, hospital trauma center data highlights that occupational injury patterns are severe and distinct, necessitating targeted safety protocols across industries (21).

Based on these findings, the specific accident characteristics and corresponding curriculum recommendations for each sector are synthesized in Table 2.

**Table 2:** Analysis of sectoral accident characteristics and implications for VET curricula.

Sector	Dominant accident types	High-risk groups	Recommended curriculum module
Construction	Falls from heights (32%); being struck by objects; electrocution.	Contract workers; general laborers.	VR-based simulations: working at heights, scaffolding safety, and fall protection systems.
Mining and industry	Machinery entrapment; crushing; explosions/fires.	Workers in small firms (<50 employees); Older workers.	Technical lockout/tagout: machinery safety protocols, gas detection, and emergency evacuation drills.
Healthcare	Needle-stick injuries (high frequency); transport accidents (high severity).	Nurses (practical and registered); residents.	Ergonomics and commuting safety: safe patient handling techniques and defensive driving/commuting safety awareness. Dominant accident types: needle-stick injuries; transport accidents; psychosocial risks (burnout, stress, patient aggression). Recommended curriculum module: ergonomics and commuting safety; resilience training: stress management and de-escalation techniques for workplace violence.
General/service	Slips, trips, and falls; psychosocial stress.	New employees (<1 month tenure).	Induction simulation: “First-month survival” training focused on hazard identification and right to refuse unsafe work.

### 3.3 Vulnerability of young workers and the “first month” effect (RQ3)

The data strongly supports the hypothesis that the transition period is the most critical phase for OHS intervention.

**Figure 2:**
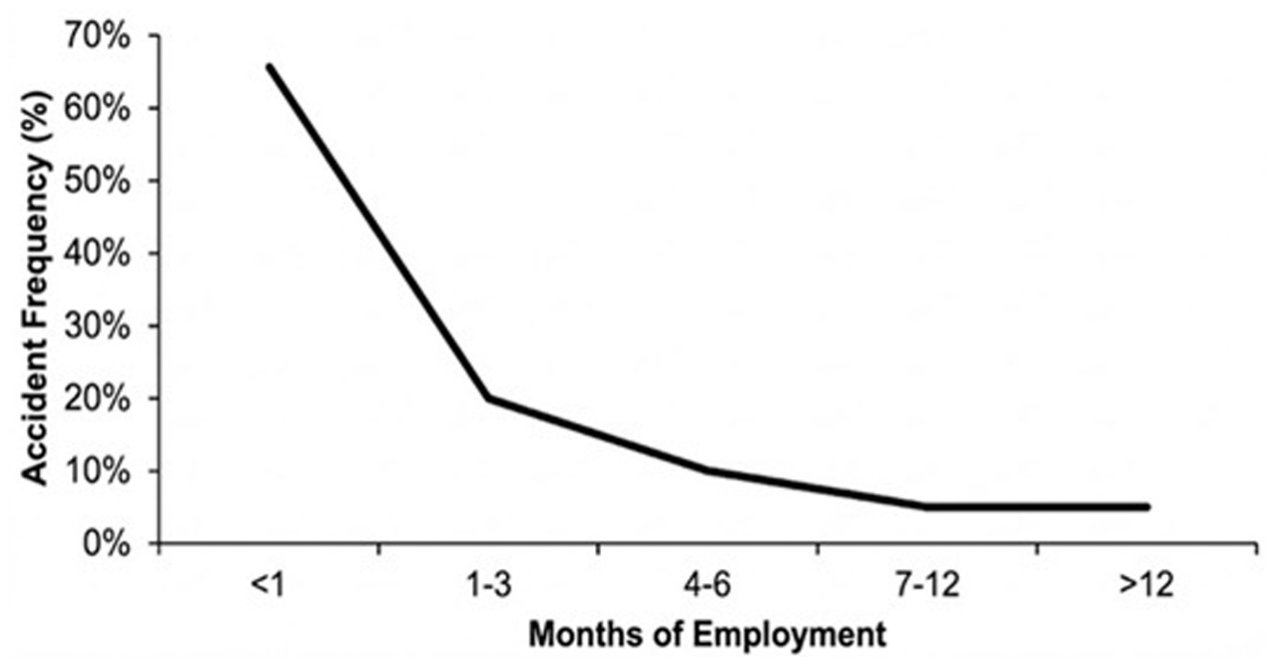
Illustrative model of the “first-month effect”: relationship between employment duration and accident frequency. This trend is primarily derived from data in high-risk industrial sectors and serves to highlight the critical vulnerability of the induction period.

- *The first month:* Multiple studies pinpoint the initial period of employment as the highest risk zone. In Bhutanese technical institutes, “67% of all accidents occurred among workers with less than 1 month of employment” (16). Similarly, in South Korea, accident frequency was highest for workers with less than 1 month of experience (22). This sharp peak in accident frequency during the induction period and its subsequent decline is visually represented in Figure 2.
- *Age vs. experience:* While older workers may suffer more severe injuries due to physical decline (1), young workers (18–24 age group) have a higher *frequency* of non-fatal injuries due to inexperience and lack of safety skills (2). Salminen’s review confirms that young workers have a higher injury rate than older workers in most countries studied (2).
- *The role of mentorship:* Qualitative research from Canada suggests that “teaching” safety rules is not the same as “learning” to work safely. Apprentices often side-step safety rules to meet production demands unless they are guided by experienced co-workers who can demonstrate how to balance safety with productivity (6).

### 3.4 Macro-factors: culture, firm size and economy

- *Firm size:* Smaller firms often lack the resources for comprehensive OHS training. Studies in Italy confirm a relationship between firm size and injury rates, with smaller firms often exhibiting higher risks (23). In Spain, mining accidents were also correlated with company size (1).
- *Economic development:* A longitudinal study in Iran found a significant relationship between the Human Development Index (HDI) and occupational accidents, suggesting that as a country’s education and economic levels rise, safety performance improves (24). Similarly, economic analysis in China revealed a negative correlation between GDP growth and fatal workplace incidents (25).
- *Safety culture:* In Brazil, the introduction of the “Accident Prevention Factor” policy led to a reduction in accidents, but continuous education is needed to sustain this culture (26). A global meta-analysis on PPE usage indicated that safety culture and regulations significantly influence whether workers use protective equipment (27).

## 4 Discussion

### 4.1 Beyond compliance: the need for active OHS pedagogy

The findings of this review strongly validate the hypothesis that OHS education is not merely a regulatory formality but a critical preventive tool. The statistical evidence from France showing a 50% reduction in injury rates among educated apprentices serves as a benchmark for the efficacy of early intervention (3). However, the qualitative data suggests that the *method* of delivery is as important as the *content*. Traditional, lecture-based safety training often fails to translate into safe behavior in high-pressure work environments (7). In contrast, models like “ZeroSicks” in Indonesia (12) and “Attitude to Work” in Finland (4) succeed because they engage students in active problem-solving and internalize safety as a value rather than a rule. Comparative studies further support this, showing that different training methods in vocational schools can lead to varying levels of safety competence, with active engagement yielding better results (28). This aligns with Laberge et al. (6) observation that “learning” in the workplace is a dynamic, social process that requires students to develop self-regulation strategies rather than just obedience to rules. A comparative analysis of these pedagogical approaches, contrasting passive instruction with active learning models, is outlined in Table 3.

**Table 3:** Comparison of OHS pedagogical approaches in VET settings.

Pedagogical approach	Methods used	Impact on safety competence	Study reference
Passive learning	Lectures, reading regulations, watching videos.	Low retention; compliance-based behavior; limited impact on “safety motivation.”	Talib et al. (7); El-Marakby et al. (17)
Active learning	“ZeroSicks” model; Problem-based scenarios; Peer discussions.	High engagement; improved internal locus of control; Better hazard recognition.	Ismara et al. (12); Nykänen et al. (4)
Situated learning	Workplace mentorship; apprenticeship with experienced peers.	Development of self-regulation strategies; adaptation to real-world constraints.	Laberge et al. (6)
Simulation	VR (Virtual reality); role-playing emergency response.	High skill transfer; emotional preparedness for high-stress situations.	Recommended in conclusion (Section 5.2)

### 4.2 The “one-size-fits-all” fallacy *vs.* sectoral reality

A significant disconnect identified in this study is the generic nature of many VET OHS curricula versus the highly specific risks of different industries. While a generic curriculum might cover basic PPE usage, it fails to prepare a healthcare student for the high-severity risk of commuting accidents (9) or a construction student for the specific dynamics of falling from heights, which accounts for nearly a third of fatalities in that sector (5, 8). The Bayesian network analysis in the mining sector highlights that training is the single most modifiable factor affecting accident severity (10). Therefore, educational institutions must shift from generic safety modules to “sector-simulated” environments where students practice responding to the specific lethal risks of their future trade.

### 4.3 Bridging the “first month” vulnerability gap

Perhaps the most critical finding for educators is the extreme vulnerability of workers during their first month of employment (16, 22). This “first-month effect” indicates that the transition from school to work is abrupt and dangerous. VET institutions often view their responsibility as ending upon graduation, while employers assume graduates are “job-ready.” This gap leaves young workers exposed. The data suggests that OHS education must include a specific “induction phase” or “transition simulation” that mimics the high-stress, low-supervision environment of the first weeks of employment, empowering students to refuse unsafe work or identify hazards independently (2, 22).

### 4.4 The challenge of small enterprises

The data consistently shows that smaller firms have higher accident rates and lower safety standards (1, 23). This poses a unique challenge for VET educators: students placed in internships or jobs in Small and Medium Enterprises (SMEs) are less likely to receive adequate on-the-job safety training than those in large corporations. Therefore, schools must over-compensate for this deficit by ensuring students bound for SMEs have a higher level of self-reliance and autonomous safety decision-making skills (11).

### 4.5 Limitations and potential bias

Although this review followed a systematic protocol, several limitations should be acknowledged. First, publication bias may be present, as studies with positive outcomes are more likely to appear in indexed journals. Second, language bias may occur, since only articles written in English were included, potentially excluding regionally important studies published in other languages. Third, regional heterogeneity in data collection and sector definitions may limit comparability across countries. Finally, the absence of meta-analytic statistical synthesis due to methodological diversity restricts causal inference. Therefore, results should be interpreted carefully and primarily used for educational and policy implications rather than direct causal assumptions.

## 5 Conclusion, recommendations and future directions

### 5.1 Conclusion

This systematic review of 32 international studies confirms that OHS education is a cornerstone of occupational safety, capable of significantly reducing injury rates among young workers. However, the current educational landscape is fragmented. While innovative models like ZeroSicks demonstrate the power of active learning, many curricula remain disconnected from the specific realities of high-risk sectors like mining and construction. The evidence highlights a critical “danger zone” during the first month of employment, necessitating a pedagogical shift from passive knowledge transfer to active competency building. Ultimately, sustainable safety is not achieved by legislation alone but by cultivating a “safety culture” that begins in the classroom and transitions seamlessly to the workplace (26, 29).

### 5.2 Recommendations

Based on the synthesized evidence, the following recommendations are proposed for educational institutions, policymakers, and industry stakeholders:

1. *Implement “Transition simulations” in curricula:* To address the high accident rates in the first month of employment (16, 22), VET institutions should introduce mandatory “induction simulations” in the final semester. These should mimic the pressure and lack of supervision of a real workplace, testing students’ ability to identify hazards under stress.
2. *Sector-specific OHS modules:* Move away from generic OHS courses.

- *For construction*: Focus intensively on “working at heights” and “scaffolding safety” using VR or physical simulations (5, 8, 18).
- *For healthcare*: Integrate modules on “safe commuting/transportation” and “ergonomics for patient handling” alongside standard biological hazard training (9).
- *For mining/industry*: Emphasize machinery lockout/tagout procedures and risk assessment matrices (1, 10).

3. *Adoption of active learning models:* Replace passive lectures with participatory models like “ZeroSicks” or “Attitude to Work” that have statistically proven efficacy in improving safety motivation and locus of control (4, 12). Evaluating the learning outcomes of these models regularly is also essential (30).
4. *Special focus on SME placements:* Schools must identify students entering Small and Medium Enterprises (SMEs) for internships and provide them with additional “self-defense” OHS training, as they are unlikely to receive robust safety onboarding from their employers (1, 23).
5. *Data-driven curriculum updates:* Educational boards should establish a feedback loop where national accident statistics (e.g., rise in specific injury types like falls or electrocution) directly trigger updates in VET OHS curricula (25, 31). Furthermore, policy interventions must be developed to identify and support high-risk groups to prevent industrial accidents effectively (32).

### 5.3 Implications for future research

Future research should focus on longitudinal designs following students during their transition from school to work in order to better understand the first-month vulnerability. Comparative analyzes between countries, sectors, and educational models will help clarify which pedagogical approaches are most effective for reducing severe accidents. Moreover, experimental designs implementing simulation-based safety modules (VR, workplace simulations) are needed to evaluate the long-term impact of active learning strategies on real injury outcomes.
